# Supplementary material for: The Antimicrobial Properties of Poplar and Aspen–Poplar Propolises and Their Active Components against Selected Microorganisms, including Helicobacter pylori
Source: Pathogens. 2022 Jan 31;11(2):191. doi: 10.3390/pathogens11020191 (PMC8875431; doi:10.3390/pathogens11020191)
Supplement: Supplementary file 1 [file pathogens-11-00191-s001.zip › pathogens-1536010-SM.pdf]

Article

The Antimicrobial Properties of Poplar and Aspen–Poplar Propolises and their Active Components Against Selected Microorganisms, Including *Helicobacter pylori*

Jarosław Widelski [1,*,†], Piotr Okińczyc [2,*,†], Emil Paluch [3], Tomasz Mroczek [4], Jakub Szperlik [5], Magdalena Żuk [6], Zbigniew Sroka [2], Zuriyadda Sakipova [7], Ioanna Chinou [8], Krystyna Skalicka-Woźniak [4], Anna Malm [9] and Izabela Korona-Głowniak [9]

Table S1. Model 1—components vs. MIC, impact of components on antibacterial activity.

**Microrganism/ Component**	***S. aureus* ATCC 25923**	***S. aureus* ATCC 29213**	***S. epidermidis* ATCC 12228**	***E. faecalis* ATCC 29212**	***M. luteus* ATCC 10240**	***B. cereus* ATCC 10876**	***H. pylori* ATCC 43504**	***C. albicans* ATCC 102231**	***C. parapsilosis* ATCC 22019**	***C. glabrata* ATCC 90030**
Caffeic acid	R=-0.053	R=0.012	R=-0.226	R=-0.313	R=-0.050	R=-0.361	R=0.061	R=-0.095	R=-0.125	R=-0.576
	p=0.883	*p*=0.973	*p*=0.531	*p*=0.378	*p*=0.891	*p*=0.305	*p*=0.867	*p*=0.795	*p*=0.730	*p*=0.081
p-Coumaric acid	R=0.871	R=0.801	R=0.923	R=0.610	R=0.855	R=0.852	R=0.728	R=0.845	R=0.869	R=0.949
	p=0.001	*p*=0.005	*p*=0.000	*p*=0.061	*p*=0.002	*p*=0.002	*p*=0.017	*p*=0.002	*p*=0.001	*p*=0.000
Vanillin	R=0.965	R=0.946	R=0.990	R=0.680	R=0.966	R=0.934	R=0.832	R=0.962	R=0.967	R=0.882
	p=0.000	*p*=0.000	*p*=0.000	*p*=0.031	*p*=0.000	*p*=0.000	*p*=0.003	*p*=0.000	*p*=0.000	*p*=0.001
Ferulic acid	R=0.842	R=0.811	R=0.840	R=0.545	R=0.852	R=0.836	R=0.669	R=0.841	R=0.859	R=0.777
	p=0.002	*p*=0.004	*p*=0.002	*p*=0.103	*p*=0.002	*p*=0.003	*p*=0.034	*p*=0.002	*p*=0.001	*p*=0.008
Isoferulic acid	R=-0.178	R=-0.170	R=-0.332	R=-0.325	R=-0.187	R=-0.430	R=-0.130	R=-0.257	R=-0.271	R=-0.584
	p=0.623	*p*=0.638	*p*=0.349	*p*=0.359	*p*=0.605	*p*=0.215	*p*=0.720	*p*=0.474	*p*=0.448	*p*=0.076
Benzoic acid	R=0.667	R=0.618	R=0.781	R=0.467	R=0.680	R=0.825	R=0.465	R=0.660	R=0.691	R=0.866
	p=0.035	*p*=0.057	*p*=0.008	*p*=0.174	*p*=0.031	*p*=0.003	*p*=0.176	*p*=0.038	*p*=0.027	*p*=0.001
3,4-Dimethylcaffeic acid (DMCA)	R=0.127	R=0.139	R=-0.026	R=0.013	R=0.113	R=-0.168	R=0.279	R=0.150	R=0.132	R=-0.159
	p=0.726	*p*=0.701	*p*=0.942	*p*=0.972	*p*=0.755	*p*=0.643	*p*=0.435	*p*=0.679	*p*=0.716	*p*=0.660
Quercetin	R=-0.354	R=-0.229	R=-0.432	R=-0.468	R=-0.342	R=-0.526	R=-0.108	R=-0.309	R=-0.352	R=-0.602
	p=0.316	*p*=0.525	*p*=0.213	*p*=0.173	*p*=0.334	*p*=0.119	*p*=0.766	*p*=0.386	*p*=0.319	*p*=0.065

Pinobanksin-5-methyl ether	R=-0.583	R=-0.447	R=-0.599	R=-0.728	R=-0.550	R=-0.677	R=-0.436	R=-0.537	R=-0.594	R=-0.801
	p=0.077	*p*=0.195	*p*=0.067	*p*=0.017	*p*=0.100	*p*=0.032	*p*=0.208	*p*=0.110	*p*=0.070	*p*=0.005
Cinnamic acid	R=0.929	R=0.944	R=0.838	R=0.571	R=0.937	R=0.755	R=0.877	R=0.931	R=0.920	R=0.590
	p=0.000	*p*=0.000	*p*=0.002	*p*=0.085	*p*=0.000	*p*=0.012	*p*=0.001	*p*=0.000	*p*=0.000	*p*=0.073
Quercetin-3-methyl ether	R=-0.481	R=-0.337	R=-0.548	R=-0.624	R=-0.452	R=-0.660	R=-0.244	R=-0.407	R=-0.464	R=-0.758
	p=0.159	*p*=0.342	*p*=0.101	*p*=0.054	*p*=0.189	*p*=0.038	*p*=0.497	*p*=0.243	*p*=0.177	*p*=0.011
Pinobanksin	R=-0.554	R=-0.454	R=-0.614	R=-0.574	R=-0.534	R=-0.621	R=-0.430	R=-0.557	R=-0.591	R=-0.798
	p=0.097	*p*=0.187	*p*=0.059	*p*=0.083	*p*=0.112	*p*=0.055	*p*=0.215	*p*=0.095	*p*=0.072	*p*=0.006
Apigenin	R=0.006	R=-0.063	R=0.047	R=0.092	R=0.005	R=0.122	R=-0.219	-R=0.071	R=-0.062	R=0.024
	p=0.987	*p*=0.862	*p*=0.898	*p*=0.801	*p*=0.989	*p*=0.738	*p*=0.542	*p*=0.845	*p*=0.866	*p*=0.947
Kempferol	R=-0.025	R=-0.078	R=-0.015	R=0.367	R=-0.052	R=0.127	R=-0.058	R=-0.071	R=-0.048	R=0.101
	p=0.946	*p*=0.831	*p*=0.968	*p*=0.297	*p*=0.887	*p*=0.727	*p*=0.874	*p*=0.845	*p*=0.895	*p*=0.781
Isorhamnetin	R=-0.271	R=0.324	R=-0.246	R=0.346	R=-0.289	R=-0.022	R=-0.265	R=-0.261	R=-0.234	R=0.003
	p=0.448	*p*=0.361	*p*=0.493	*p*=0.328	*p*=0.418	*p*=0.953	*p*=0.459	*p*=0.467	*p*=0.516	*p*=0.993
Luteolin-5-methyl ether	R=-0.505	R=-0.460	R=-0.529	R=-0.006	R=-0.487	R=-0.344	R=-0.430	R=-0.469	R=-0.483	R=-0.535
	p=0.136	*p*=0.181	*p*=0.116	*p*=0.987	*p*=0.153	*p*=0.330	*p*=0.215	*p*=0.172	*p*=0.157	*p*=0.111
Quercetine-5,7-dimethyl ether	R=-0.156	R=-0.163	R=-0.173	R=0.331	R=-0.164	R=-0.049	R=-0.076	R=-0.111	R=-0.111	R=-0.076
	p=0.668	*p*=0.653	*p*=0.632	*p*=0.350	*p*=0.651	*p*=0.894	*p*=0.834	*p*=0.761	*p*=0.760	*p*=0.834
Rhamnetin	R=-0.318	R=-0.239	R=-0.392	R=-0.279	R=-0.308	R=-0.364	R=-0.203	R=-0.326	R=-0.346	R=-0.543
	p=0.371	*p*=0.505	*p*=0.262	*p*=0.436	*p*=0.387	*p*=0.301	*p*=0.574	*p*=0.359	*p*=0.328	*p*=0.105
Quercetin-dimethyl ether	R=-0.243	R=-0.197	R=-0.303	R=0.004	R=-0.242	R=-0.296	R=-0.071	R=-0.167	R=-0.192	R=-0.303
	p=0.499	*p*=0.585	*p*=0.394	*p*=0.991	*p*=0.500	*p*=0.407	*p*=0.845	*p*=0.645	*p*=0.595	*p*=0.395
2-Acetyl-1-caffeoyl-3-*p*-coumaroylglycerol	R=0.485	R=0.442	R=0.453	R=0.115	R=0.483	R=0.384	R=0.365	R=0.483	R=0.504	R=0.466
	p=0.156	*p*=0.201	*p*=0.188	*p*=0.752	*p*=0.157	*p*=0.274	*p*=0.300	*p*=0.157	*p*=0.138	*p*=0.175
Quercetin-dimethyl ether	R=-0.465	R=-0.364	R=-0.504	R=-0.499	R=-0.450	R=-0.609	R=-0.266	R=-0.397	R=-0.443	R=-0.625
	p=0.175	*p*=0.301	*p*=0.137	*p*=0.142	*p*=0.191	*p*=0.062	*p*=0.458	*p*=0.256	*p*=0.199	*p*=0.053
2-Acetyl-3-caffeoyl-1-feruloylglycerol	R=0.536	R=0.485	R=0.574	R=0.217	R=0.545	R=0.560	R=0.362	R=0.537	R=0.573	R=0.641
	p=0.110	*p*=0.155	*p*=0.083	*p*=0.547	*p*=0.103	*p*=0.092	*p*=0.305	*p*=0.109	*p*=0.083	*p*=0.046
Caffeic acid prenyl ester 1	R=-0.287	R=-0.297	R=-0.339	R=-0.405	R=-0.317	R=-0.438	R=-0.426	R=-0.375	R=-0.382	R=-0.520
	p=0.422	*p*=0.404	*p*=0.338	*p*=0.246	*p*=0.372	*p*=0.206	*p*=0.219	*p*=0.286	*p*=0.276	*p*=0.123
Chrysin	R=-0.585	R=-0.444	R=-0.663	R=-0.652	R=-0.555	R=-0.741	R=-0.315	R=-0.524	R=-0.580	R=-0.858
	p=0.076	*p*=0.199	*p*=0.037	*p*=0.041	*p*=0.096	*p*=0.014	*p*=0.375	*p*=0.120	*p*=0.079	*p*=0.002
Pinocembrin	R=-0.477	R=-0.381	R=-0.458	R=-0.508	R=-0.450	R=-0.416	R=-0.444	R=-0.454	R=-0.484	R=-0.545
	p=0.164	*p*=0.278	*p*=0.184	*p*=0.133	*p*=0.192	*p*=0.231	*p*=0.198	*p*=0.187	*p*=0.156	*p*=0.103
	R=0.056	R=-0.063	R=0.112	R=0.600	R=0.027	R=0.319	R=-0.067	R=0.023	R=0.067	R=0.387

Acacetin	*p*=0.878	*p*=0.862	*p*=0.757	*p*=0.067	*p*=0.941	*p*=0.368	*p*=0.854	*p*=0.949	*p*=0.854	*p*=0.269
Caffeic acid prenyl ester 2	R=-0.225	R=-0.156	R=-0.255	R=-0.214	R=-0.215	R=-0.336	R=-0.047	R=-0.143	R=-0.178	R=-0.298
	p=0.532	*p*=0.668	*p*=0.478	*p*=0.553	*p*=0.550	*p*=0.343	*p*=0.897	*p*=0.694	*p*=0.622	*p*=0.403
Pinocembrin chalcone	R=-0.225	R=-0.156	R=-0.255	R=-0.214	R=-0.215	R=-0.336	R=-0.047	R=-0.143	R=-0.178	R=-0.298
	p=0.532	*p*=0.668	*p*=0.478	*p*=0.553	*p*=0.550	*p*=0.343	*p*=0.897	*p*=0.694	*p*=0.622	*p*=0.403
*Caffeic acid prenyl ester 3	R=-0.288	R=-0.269	R=-0.399	R=-0.393	R=-0.306	R=-0.579	R=-0.069	R=-0.285	R=-0.305	R=-0.476
	p=0.420	*p*=0.452	*p*=0.254	*p*=0.261	*p*=0.390	*p*=0.080	*p*=0.851	*p*=0.425	*p*=0.392	*p*=0.165
*Caffeic acid benzyl ester	R=0.153	R=0.184	R=0.021	R=-0.179	R=0.155	R=-0.071	R=0.161	R=0.114	R=0.106	R=-0.214
	p=0.673	*p*=0.610	*p*=0.955	*p*=0.620	*p*=0.668	*p*=0.846	*p*=0.657	*p*=0.755	*p*=0.771	*p*=0.553
Sakuranetin	R=-0.011	R=-0.110	R=0.091	R=0.501	R=-0.034	R=0.281	R=-0.111	R=-0.018	R=0.018	R=0.401
	p=0.976	*p*=0.763	*p*=0.803	*p*=0.141	*p*=0.927	*p*=0.431	*p*=0.761	*p*=0.961	*p*=0.962	*p*=0.250
Genkwanin	R=0.054	R=-0.061	R=0.090	R=0.622	R=0.026	R=0.309	R=-0.065	R=0.022	R=0.065	R=0.342
	p=0.882	*p*=0.867	*p*=0.806	*p*=0.055	*p*=0.943	*p*=0.385	*p*=0.859	*p*=0.951	*p*=0.859	*p*=0.334
Galangin	R=-0.550	R=-0.461	R=-0.581	R=-0.712	R=-0.529	R=-0.638	R=-0.458	R=-0.564	R=-0.594	R=-0.751
	p=0.100	*p*=0.180	*p*=0.078	*p*=0.021	*p*=0.116	*p*=0.047	*p*=0.183	*p*=0.090	*p*=0.070	*p*=0.012
Kaempheride	R=0.155	R=0.019	R=0.274	R=0.540	R=0.123	R=0.426	R=-0.007	R=0.116	R=0.169	R=0.611
	p=0.670	*p*=0.959	*p*=0.444	*p*=0.108	*p*=0.736	*p*=0.220	*p*=0.984	*p*=0.749	*p*=0.640	*p*=0.061
2-Acetyl-1,3-di-*p*-Coumaroyl glycerol	R=0.808	R=0.713	R=0.782	R=0.900	R=0.789	R=0.868	R=0.640	R=0.783	R=0.819	R=0.829
	p=0.005	*p*=0.021	*p*=0.008	*p*=0.000	*p*=0.007	*p*=0.001	*p*=0.046	*p*=0.007	*p*=0.004	*p*=0.003
Pinobanksin-3O-acetate	R=-0.627	R=-0.516	R=-0.631	R=-0.687	R=-0.589	R=-0.617	R=-0.546	R=-0.603	R=-0.637	R=-0.775
	p=0.053	*p*=0.127	*p*=0.050	*p*=0.028	*p*=0.073	*p*=0.057	*p*=0.102	*p*=0.065	*p*=0.048	*p*=0.008
Quercetin-dimethyl	R=0.057	R=-0.065	R=0.127	R=0.582	R=0.028	R=0.325	R=-0.068	R=0.024	R=0.069	R=0.416
	p=0.875	*p*=0.859	*p*=0.726	*p*=0.077	*p*=0.939	*p*=0.359	*p*=0.851	*p*=0.948	*p*=0.851	*p*=0.232
**2-Acetyl-3-*p*-coumaroyl-1-feruloylglycerol	R=0.833	R=0.765	R=0.811	R=0.645	R=0.827	R=0.819	R=0.658	R=0.824	R=0.853	R=0.812
	p=0.003	*p*=0.010	*p*=0.004	*p*=0.044	*p*=0.003	*p*=0.004	*p*=0.039	*p*=0.003	*p*=0.002	*p*=0.004

Table legend: R—square R parameter; *p*—Pearson's correlation parameter; *S. aureus—Staphylococcus aureus; S. epidermidis—Staphylococcus epidermidis; E. faecalis—Enterococcus faecalis; M. luteus—Micrococcus luteus; B. cereus—Bacillus cereus; H. pylori—Helicobacter pylori; C. albicans—Candida albicans; C. parapsilosis—Candida parapsilosis; C. glabrata—Candida glabrata.*

Table S2. Model 2—significant components vs. significant components, an analysis of connections between presence of significant components in Model 1.

Significant Component	*p*-Coumaric Acid	Vanilline	Ferulic Acid	Benzoic Acid	Pinobanksin-5-Methyl Ether	Cinnamic Acid	Pinobanksin	2-Acetyl-3-Caffeoyl-1-Feruloylglycerol	Chrysin	Galangin	2-Acetyl-1,3-di-*p*-Coumaroylglycerol	Pinobanksin-3O-Acetate	**2-Acetyl-3-*p*-Coumaroyl-1-Feruloylglycerol
p-Coumaric acid	R=1.000	R=0.938	R=0.839	R=0.803	R=-0.768	R=0.761	R=-0.732	R=0.679	R=-0.784	R=-0.667	R=0.819	R=-0.783	R=0.871
	p= ---	*p*=0.000	*p*=0.002	*p*=0.005	*p*=0.009	*p*=0.011	*p*=0.016	*p*=0.031	*p*=0.007	*p*=0.035	*p*=0.004	*p*=0.007	*p*=0.001
Vanillin	R=0.938	R=1.000	R=0.870	R=0.790	R=-0.599	R=0.874	R=-0.610	R=0.616	R=-0.639	R=-0.572	R=0.776	R=-0.633	R=0.837
	p=0.000	*p*= ---	*p*=0.001	*p*=0.007	*p*=0.067	*p*=0.001	*p*=0.061	*p*=0.058	*p*=0.047	*p*=0.084	*p*=0.008	*p*=0.049	*p*=0.002
Ferulic acid	R=0.839	R=0.870	R=1.000	R=0.873	R=-0.592	R=0.845	R=-0.531	R=0.886	R=-0.685	R=-0.440	R=0.751	R=-0.480	R=0.952
	p=0.002	*p*=0.001	*p*= ---	*p*=0.001	*p*=0.071	*p*=0.002	*p*=0.114	*p*=0.001	*p*=0.029	*p*=0.203	*p*=0.012	*p*=0.160	*p*=0.000
Benzoic acid	R=0.803	R=0.790	R=0.873	R=1.000	R=-0.613	R=0.555	R=-0.529	R=0.814	R=-0.797	R=-0.431	R=0.672	R=-0.413	R=0.791
	p=0.005	*p*=0.007	*p*=0.001	*p*= ---	*p*=0.060	*p*=0.096	*p*=0.116	*p*=0.004	*p*=0.006	*p*=0.214	*p*=0.033	*p*=0.236	*p*=0.006
Pinobanksin-5-methyl ether	R=-0.768	R=-0.599	R=-0.592	R=-0.613	R=1.000	R=-0.389	R=0.771	R=-0.517	R=0.928	R=0.804	R=-0.917	R=0.883	R=-0.764
	p=0.009	*p*=0.067	*p*=0.071	*p*=0.060	*p*= ---	*p*=0.266	*p*=0.009	*p*=0.126	*p*=0.000	*p*=0.005	*p*=0.000	*p*=0.001	*p*=0.010
Cinnamic acid	R=0.761	R=0.874	R=0.845	R=0.555	R=-0.389	R=1.000	R=-0.383	R=0.577	R=-0.370	R=-0.343	R=0.642	R=-0.457	R=0.801
	p=0.011	*p*=0.001	*p*=0.002	*p*=0.096	*p*=0.266	*p*= ---	*p*=0.275	*p*=0.081	*p*=0.293	*p*=0.332	*p*=0.046	*p*=0.184	*p*=0.005
Pinobanksin	R=-0.732	R=-0.610	R=-0.531	R=-0.529	R=0.771	R=-0.383	R=1.000	R=-0.514	R=0.701	R=0.954	R=-0.767	R=0.893	R=-0.705
	p=0.016	*p*=0.061	*p*=0.114	*p*=0.116	*p*=0.009	*p*=0.275	*p*= ---	*p*=0.128	*p*=0.024	*p*=0.000	*p*=0.010	*p*=0.000	*p*=0.023
2-Acetyl-3-caffeoyl-1-feruloylglycerol	R=0.679	R=0.616	R=0.886	R=0.814	R=-0.517	R=0.577	R=-0.514	R=1.000	R=-0.640	R=-0.347	R=0.552	R=-0.360	R=0.855
	p=0.031	*p*=0.058	*p*=0.001	*p*=0.004	*p*=0.126	*p*=0.081	*p*=0.128	*p*= ---	*p*=0.046	*p*=0.326	*p*=0.098	*p*=0.307	*p*=0.002
Chrysin	R=-0.784	R=-0.639	R=-0.685	R=-0.797	R=0.928	R=-0.370	R=0.701	R=-0.640	R=1.000	R=0.673	R=-0.862	R=0.722	R=-0.775
	p=0.007	*p*=0.047	*p*=0.029	*p*=0.006	*p*=0.000	*p*=0.293	*p*=0.024	*p*=0.046	*p*= ---	*p*=0.033	*p*=0.001	*p*=0.018	*p*=0.008
Galangin	R=-0.667	R=-0.572	R=-0.440	R=-0.431	R=0.804	R=-0.343	R=0.954	R=-0.347	R=0.673	R=1.000	R=-0.815	R=0.925	R=-0.642
	p=0.035	*p*=0.084	*p*=0.203	*p*=0.214	*p*=0.005	*p*=0.332	*p*=0.000	*p*=0.326	*p*=0.033	*p*= ---	*p*=0.004	*p*=0.000	*p*=0.045
2-Acetyl-1,3-di-*p*-coumaroylglycerol	R=0.819	R=0.776	R=0.751	R=0.672	R=-0.917	R=0.642	R=-0.767	R=0.552	R=-0.862	R=-0.815	R=1.000	R=-0.844	R=0.874
	p=0.004	*p*=0.008	*p*=0.012	*p*=.033	*p*=0.000	*p*=0.046	*p*=0.010	*p*=0.098	*p*=0.001	*p*=0.004	*p*= ---	*p*=0.002	*p*=0.001
Pinobanksin-3O-acetate	R=-0.783	R=-0.633	R=-0.480	R=-0.413	R=0.883	R=-0.457	R=0.893	R=-0.360	R=0.722	R=0.925	R=-0.844	R=1.000	R=-0.692
	p=0.007	*p*=0.049	*p*=0.160	*p*=0.236	*p*=0.001	*p*=0.184	*p*=0.000	*p*=0.307	*p*=0.018	*p*=0.000	*p*=0.002	*p*= ---	*p*=0.027
	R=0.871	R=0.837	R=0.952	R=0.791	R=-0.764	R=0.801	R=-0.705	R=0.855	R=-0.775	R=-0.642	R=0.874	R=-0.692	R=1.000

**2-Acetyl-3-*p*-coumaroyl-1-feruloylglycerol	p=0.001	p=0.002	p=0.000	p=0.006	p=0.010	p=0.005	p=0.023	p=0.002	p=0.008	p=0.045	p=0.001	p=0.027	p= ---

Table legend: R—square R parameter; *p*—Pearson's correlation parameter.

Table S3. The antimicrobial activity (mg/L) of reference drugs: vancomycin, clarithromycin, ciprofloxacin, and nystatin against reference strains.

Microorganism	MIC [mg/L]
Gram-Positive Bacteria	**Vancomycin**
S. aureus ATCC 25923	0.49
S. aureus ATCC 29213	0.98
S. epidermidis ATCC 12228	0.98
E. faecalis ATCC 29212	1.95
M. luteus ATCC 10240	0.12
B. cereus ATCC 10876	0.98
Gram-Negative Microaerophilic Bacteria	**Clarithromycin**
H. pylori ATCC 43504	0.125
Gram-Negative Bacteria	**Ciprofloxacin**
S. typhimurium ATCC 14028	0.061
E. coli ATCC 25922	0.015
P. mirabilis ATCC 12453	0.03
K. pneumoniae ATCC 13883	0.12
P. aeruginosa ATCC 9027	0.48
Yeasts	**Nystatin**
C. albicans ATCC 102231	0.48
C. parapsilosis ATCC 22019	0.24
C. glabrata ATCC 90030	0.24

Table S4. Input data of statistical analyses.

Microorganism—Mic (mg/ML)	PLS1	PLS2	PLS3	PLU	UK1	UK2	UK3	UKT	KZ.AO	G.PA
S. aureus ATCC 25923	0.16	0.63	0.08	0.16	0.04	0.04	0.08	0.01	0.16	0.02
S. aureus ATCC 29213	0.31	2.50	0.08	0.31	0.16	0.16	0.16	0.01	0.31	0.08
S. epidermidis ATCC	0.08	0.31	0.04	0.16	0.04	0.04	0.08	0.01	0.08	0.02
E. faecalis ATCC 29212	0.31	2.50	0.16	0.63	0.16	0.16	0.16	0.08	2.50	0.08
M. luteus ATCC 10240	0.08	0.31	0.04	0.08	0.04	0.04	0.04	0.01	0.08	0.02
B. cereus ATCC 10876	0.16	0.63	0.04	0.31	0.16	0.16	0.16	0.02	0.31	0.02
C. albicans ATCC 102231	0.31	1.25	0.08	0.31	0.16	0.16	0.16	0.08	0.31	0.16
C. parapsilosis ATCC 22019	0.31	1.25	0.04	0.31	0.08	0.16	0.08	0.02	0.31	0.08
C. glabrata ATCC 90030	0.31	0.63	0.08	0.63	0.16	0.16	0.16	0.08	0.31	0.08
Components (%PA)	**PLS1**	**PLS2**	**PLS3**	**PLU**	**UK1**	**UK2**	**UK3**	**UKT**	**KZ.AO**	**G.PA**
Caffeic acid	1.87	2.01	3.08	0.40	1.85	1.83	1.95	2.17	1.01	1.66
p-Coumaric acid	6.20	12.10	2.08	9.01	1.16	1.37	1.20	1.47	3.23	0.41
Vanilline	0.68	2.92	0.12	1.28	0.26	0.28	0.29	0.00	0.43	0.00
Ferulic acid	4.54	6.29	0.98	3.03	2.57	2.66	2.18	0.95	2.12	0.48
Isoferulic acid	1.15	0.76	2.14	0.00	1.03	0.86	1.07	0.94	0.58	0.67
Benzoic acid	1.68	2.50	0.53	2.35	1.50	1.64	1.27	0.53	1.23	0.13
3,4-Dimethylcaffeic acid (DMCA)	1.37	1.03	1.05	0.00	0.08	0.09	0.08	0.55	0.52	2.13
Quercetin	0.19	0.32	0.46	0.00	0.51	0.44	0.49	1.41	0.00	0.57
Pinobanksin-5-methyl ether	0.47	0.44	1.87	0.24	2.32	1.89	2.29	1.73	0.00	2.41
Cinnamic acid	0.44	1.27	0.13	0.00	0.16	0.00	0.00	0.00	0.00	0.00
Quercetin-3-methyl ether	0.28	0.29	0.66	0.00	0.69	0.70	0.70	0.82	0.00	1.37
Pinobanksin	1.15	0.91	3.49	0.31	3.53	3.19	3.52	4.23	1.21	1.60
Apigenin	1.02	0.99	1.79	1.17	1.51	1.24	1.56	0.00	1.45	0.82
Kempferol	0.77	1.07	1.16	1.36	1.07	1.01	1.13	1.86	1.87	0.48
Isorhamnetin	0.24	0.14	0.20	0.77	0.44	0.49	0.43	0.88	1.49	0.52
Luteolin-5-methyl ether	0.39	0.53	1.00	0.70	1.14	1.21	1.13	0.86	1.56	1.41
Quercetine-5,7-dimethyl ether	0.15	0.35	0.35	0.57	0.31	0.41	0.35	0.44	1.20	1.19
Rhamnetin	0.19	0.31	0.63	0.00	0.69	0.63	0.67	1.29	0.36	0.00
Quercetin-dimethyl ether	0.00	0.00	0.00	0.00	0.00	0.00	0.00	0.15	0.39	1.01
2-Acetyl-1-caffeoyl-3-*p*-coumaroylglycerol	1.65	1.00	0.00	0.42	0.38	0.38	0.30	0.25	0.00	0.00
Quercetin-dimethyl ether	0.11	0.00	0.75	0.00	0.50	0.60	0.70	0.61	0.00	2.40
2-Acetyl-3-caffeoyl-1-feruloylglycerol	1.02	0.77	0.00	0.56	0.31	0.57	0.32	0.00	0.15	0.00
Caffeic acid prenyl ester 1	1.52	0.56	3.10	0.11	0.60	1.56	3.08	2.41	0.59	0.00
Chrysin	4.46	3.87	11.81	1.11	12.68	10.39	10.23	13.38	2.86	17.47
Pinocembrin	5.41	4.88	6.52	5.10	10.61	9.48	10.68	11.40	5.13	4.52

Acacetin	0.00	0.00	0.00	4.44	0.00	0.00	0.00	0.00	8.28	0.00
Caffeic acid prenyl ester 2	0.00	0.00	0.00	0.00	0.00	0.00	0.00	0.00	0.00	4.04
Pinocembrin chalcone	0.00	0.00	0.00	0.00	0.00	0.00	0.00	0.00	0.00	1.02
*Caffeic acid prenyl ester 3	2.26	0.22	3.46	0.00	0.00	0.00	0.00	1.73	0.00	4.93
*Caffeic acid benzyl ester	5.44	4.52	4.62	0.00	3.54	3.45	3.66	5.25	0.75	0.00
Sakuranetin	0.53	0.84	0.00	7.10	1.65	1.16	1.37	1.46	9.23	2.37
Genkwanin	0.00	0.00	0.00	1.62	0.00	0.00	0.00	0.00	3.72	0.00
Galangin	5.78	3.18	12.19	2.61	11.62	10.91	11.75	11.49	1.88	5.31
Kaempheride	1.19	0.95	0.23	4.97	0.34	0.67	0.55	0.57	5.03	0.00
2-Acetyl-1,3-di-*p*-coumaroylglycerol	4.39	7.56	0.50	4.05	0.56	0.91	0.47	0.42	6.57	0.00
Pinobanksin-3O-acetate	3.83	1.62	8.89	1.77	12.32	12.18	12.18	8.87	2.47	6.68
Quercetin-dimethyl	0.00	0.00	0.00	1.03	0.00	0.00	0.00	0.00	1.69	0.00
**2-Acetyl-3-*p*-coumaroyl-1-feruloylglycerol	2.46	2.98	0.00	1.32	0.50	0.65	0.42	0.00	1.23	0.00

Table legend: * - component tenatively identified; ** - subtitution positioning of glycerol was tenatively identified; G**P**—Greece, Parga; **KZ**—Kazakhstan, Almastka Oblast; **PLS1**—Poland, Lower Silesia Region 1; **PLS2**—Poland, Lower Silesia Region 2; **PLS3**—Poland, Lower Silesia Region3; **PLU**—Poland, Lubelszczyzna Region; **UK1**—Ukraine, Khmelnitsky 1; **UK2**—Ukraine, Khmelnitsky 2; **UK3**—Ukraine, Khmelnitsky 3; **UT**—Ukraine, Tarnopol.
